# Nitrous Oxyd Rumors

**Published:** 1871-05

**Authors:** 


### NITROUS OXYD RUMORS.

The reckless and indiscriminate use of nitrous oxyd is beginning to attract attention. This is not strange. The humbugged public is proverbially patient. Mystify anything, and people will swallow it, or choke in the attempt. Dockroot tea and molasses, prepared by some genuine "old Doctor Jacob," will be drunk with greediness, and "vegetable pills" made of calomel, will be taken with a relish. Nitrous oxyd, is "laughing gas," and has been administered by fools to fools, for the amusement of fools, for three quarters of a century. The public mind has been thus educated to regard the inhalation of nitrous oxyd as a good joke; and this accounts for the fact that people will take the gas from men too ignorant to be trusted with broken wheelbarrows—that they will let men take them so far toward "kingdom come," that they cease to know the kingdom here, who scarcely know a heart from a stomach—men who are aware of no difference in the function of respiration, and the action of a blacksmith's bellows, except that in breathing, the air comes out at the same hole it went in at.

All classes of the public have been at fault. Hundreds inhale the gas every day; and yet even physicians are indifferent. All the ordinary devices are used to induce people to resort to the gas. It is daily advertised at rates below the cost of preparation. Economy must be studied. How to make a little gas accomplish a great deal, is a practical question with such operators. To have the same gas breathed over and over, is an ordinary resort of such practitioners. More than once have we been consulted as to the best and *cheapest* way to purify the gas breathed by one patient, so that it would answer for a succeeding one. We have not reached complete sanctification yet, and our answer would not look well in print.

But there are symptoms of an awakening. A rumor of a death reaches us. Then of a patient becoming (or remaining)

insane, and going to an asylum. Next of death from congestion of the brain, following the use of the gas. These rumors may be all untrue; but smoke is a sign of fire.

Now, nitrous oxyd, properly prepared, and *properly used*, is among the safest of all medicinal agents. But here comes the difficulty. At present, it is prepared by the person using it, who often is destitute of chemical knowledge. The preparation of it is difficult and complex. Few articles in the materia medica, are prepared with more difficulty, if so prepared that absolute purity is insured. The substance most frequently contaminating it is a deadly poison. When concentrated, it can not be breathed at all. When diluted, either with nitrous oxyd or atmospheric air, it may be breathed; and even when much diluted, its effects are serious, and usually grow worse for several days after taking the poison. And this poison, (nitric oxyd) is as likely to be prepared from pure, as from impure nitrate of ammonia.

In view of all these facts, it is strange that more accidents do not occur, even though many that take place are never reported. The more experienced members of our profession are usually busy, and extract but few teeth. They do not wish to be annoyed with anæsthetic practice. The consequence is that very much of the nitrous oxyd used is administered by the young and inexperienced. And it is a sad truth, that some of the experienced are none the better from their experience, but are simply confirmed in their unsound theories, and incorrect habits.

In all this, we have said nothing against our profession, as such. If each surgeon had to prepare his own chloroform, how many could do it? Yet, pure chloroform is prepared with less difficulty than pure nitrous oxyd.

Nor have we said a word against the legitimate use of nitrous oxyd. As a general rule, it ought to be used for all very severe and brief operations. Few agents more uniformly give the satisfaction we have a right to expect. But it will not do to give carbonic acid, or nitric oxyd along with it, and look for perfect success.

We hope there will soon be a proper public sentiment on the subject. Nothing short of this will correct present abuses. We may, sometime, suggest a "more excellent way." W.

